# Comparative Metabolomic Analysis of the Nutritional Aspects from Ten Cultivars of the Strawberry Fruit

**DOI:** 10.3390/foods12061153

**Published:** 2023-03-09

**Authors:** Xinlu Wang, Linxia Wu, Jing Qiu, Yongzhong Qian, Meng Wang

**Affiliations:** 1 Institute of Quality Standard and Testing Technology, Beijing Academy of Agriculture and Forestry Sciences, Beijing 100097, China; 2 Institute of Quality Standards and Testing Technology for Agro-Products, Chinese Academy of Agricultural Sciences, Key Laboratory of Agri-Food Quality and Safety, Ministry of Agriculture and Rural Affairs, Beijing 100081, China

**Keywords:** strawberry, UHPLC-Q-Exactive Orbitrap MS, metabolomics, amino acids, polyphenols

## Abstract

Strawberry (*Fragaria × ananassa*) is among the most widely cultivated fruits with good taste and rich nutrients. Many strawberry species, including white strawberries, are planted all over the world. The metabolic profiles of strawberry and distinctions among different cultivars are not fully understood. In this study, non-targeted metabolomics based on UHPLC-Q-Exactive Orbitrap MS was used to analysis the metabolites in 10 strawberry species. A total of 142 compounds were identified and were divided into six categories. Tochiotome may differ most from the white strawberry (Baiyu) by screening 72 differential metabolites. Histidine, apigenin, cyanidin 3-glucoside and peonidin 3-glucoside had potential as biomarkers for distinguishing Baiyu and another 11 strawberry groups. Amino acid metabolisms, anthocyanin biosynthesis and flavonoid biosynthesis pathways were mainly involved in the determination of the nutrition distinctions. This research contributes to the determination of the nutrition and health benefits of different strawberry species.

## 1. Introduction

Strawberry (*Fragaria × ananassa*) is among the most widely cultivated fruits, with a total of approximately 8,882,500 tons production all over the world in 2019 [1]. Strawberries have good taste and appearance and are rich in nutrients. Organic acids, amino acids, vitamins, other aromatic substances, and nutrients have been reported as the main determinants of strawberry quality and flavor [2]. For example, strawberry sweetness is determined by sugar, whereas acidity and umami are regulated by organic acids [3]. The nutrients and bioactive compounds in strawberries, such as vitamins and phenolic constituents, carry benefits and biological functions, such as antioxidation, anti-inflammatory, and anticancer [4]. The content of vitamin C was up to 58.8 mg per 100 g fresh strawberries [5]. Anthocyanins are well known phenolic constituents in strawberries, and the reported content was from 150 mg/kg to 600 mg/kg in fresh samples [6]. An increasing number of in vitro and in vivo studies have demonstrated that strawberry consumption was related to the reduction of the risks of cardiovascular disease (CVD), type 2 diabetes, neurodegeneration, and even cancers [7,8,9,10].

However, composition and contents in strawberries are different according to species, geographical origin, genetic makeup, and agronomic practices [11]. For example, recent research found that sucrose and fructo-oligo-saccharides (FOS) can be used to distinguish Mexico-grown and Canada-grown strawberries [12]. Similarly, the content of melatonin was found from 1.38 ng/g to 11.26 ng/g in four species of strawberry, namely, Camarosa, Candonga, Festival, and Primoris [5]. To better elucidate the differences in volatile compositions between Albion and Juliette varieties, researchers used comprehensive two-dimensional gas chromatography (GC × GC) combined with time-of-flight mass spectrometry (ToFMS) [13]. Ninety-four differential compounds were identified, including acidic ingredients. These results provide possible explanations for the sweeter flavor of Juliette than Albion. White strawberry is becoming popular in recent years due to its special appearance, and the white strawberry variety of “Baiyu” (BY) was semidomesticated in China with limited research. It is widely known that anthocyanins contribute significantly to the color of fruits, vegetables and flowers [14]. Therefore, the composition differences, including the disparity of anthocyanin contents between BY and other strawberry cultivars, need to be studied. Findings will provide important information for future breeding programs.

Nowadays, metabolomic analysis has been increasingly used to identify the overall components that accumulate during fruit ripening to help confirm the differences among strawberries from different genetic backgrounds, agronomic management styles, maturity stages, and growing conditions [15]. For example, a recent study explored metabolic differences in six strawberry cultivars by using metabolomics analysis [16]. The polyphenol profile in Praratchatan No.80 and fatty acid synthesis/oxidation in Akihime were critical distinctions. Interestingly, a recent study revealed the relationships between volatile compounds in strawberries and consumer acceptability through untargeted metabolomics. The quantitative results identified nine aroma biomarkers that impact consumer’s preference for strawberry preserves [17].

The present study focused on the metabolites in 12 groups of strawberry cultivars and distinguished their differences by using untargeted metabolomics analysis. The obtained results provided a metabolite profiling of 142 compounds in positive and negative modes. All of the identified compounds were divided into six categories, and the total contents of each category were analyzed. Furthermore, pairwise comparisons were conducted between the white strawberry group with other strawberry groups by using multivariate statistical analysis. This study is the first to identify and compare the metabolites in 10 strawberry cultivars. The results obtained contribute to an investigation of nutrition in different strawberry cultivars and provide a scientific basis for postharvest aspects of strawberry.

## 2. Materials and Methods

### 2.1. Sample Collection

This study included 12 groups of strawberries, with a total of 10 strawberry cultivars. The cultivar names were Baiyu (BY), Fenyu (FY), Guangdian (GD), Benihoppe (HY1, HY2, and HY3), Ssanta (SDH), Kaorino (SZ), Tongzhougongzhu (TZGZ), Yuexiu (YX), Tochiotome (YY), and Akihiime (ZJ). In particular, since the Benihoppe cultivar was widely cultivated in China, a total of three groups (HY1, HY2, and HY3) of this cultivar from the same season and region were chosen in this study. The strawberries were collected from Beijing (China) during February and March 2022. The samples were immediately frozen in liquid nitrogen after harvesting and stored at −80 °C until the extraction of metabolites.

### 2.2. Reagents

Methanol and acetonitrile (liquid-chromatography-grade) were purchased from Merck (Darmstadt, Germany). Deionized water (18.2 MΩ) was obtained from a Milli-Q water purification system (Millipore, Boston, MA, USA). Formic acid and ammonium formate were supplied by Sigma-Aldrich (St. Louis, MO, USA).

### 2.3. Sample Preparation for Metabolite Extraction

Frozen strawberry samples (1 g) were placed into 15 mL Eppendorf tubes and pre-chilled in liquid nitrogen. Then, 10 mL methanol-water (8:2, *v*/*v*) was added to the samples. After that, the mixtures were vortexed for 1 min and subjected to ultrasonic vibration for 30 min. They were then centrifuged for 5 min at 10,000 r min^−1^ at 4 °C. The supernatants were collected carefully and filtered through 0.2 µm polytetrafluoroethylene filters for analysis. Four replicates were prepared in each strawberry group. Equal quantities of the sample supernatants were mixed to prepare the quality control (QC) sample, which was embedded in the batch of every six samples during the analysis to assess the system’s stability.

### 2.4. Non-Targeted Metabolomics by Ultra-HPLC (UHPLC) Q-Exactive Orbitrap MS

Chromatographic separation was achieved with an Ultimate 3000 UHPLC system (Dionex, Boston, MA, USA) equipped with an Xbridge Amide column (2.1 mm × 150 mm × 3.5 µm, Waters) at 40 °C. The flow rate was 0.3 mL min^−1^. Mobile phase A consisted of water with 0.15% formic acid and 10 mM ammonium formate. Mobile phase B consisted of acetonitrile with 0.15% formic acid and 10 mM ammonium formate. The gradient conditions were set as follows: 90–80% B for 0–8 min; 80–70% B for 8–13 min; 70–60% B for 13–16 min; 60–90% B for 16–16.1 min; and 90% B for 16.1–20 min. The injection volume was 2 µL. The sample tray was set at 4 °C.

Mass spectrometric detection was performed on a Q-Exactive Orbitrap mass spectrometer equipped with an ESI ion source operated in the positive and negative ion modes, respectively (Thermo, Boston, MA, USA). The experimental parameters were as follows: spray voltage, 3.00 kV; sheath gas pressure, 30 psi; auxiliary gas pressure, 10 arbitrary units; capillary temperature, 320 °C; auxiliary gas heater temperature, 350 °C; scan mode, full MS (70,000 resolution)-dd/MS2 (17,500 resolution; NCE, 30 eV); and scan range, 70–1050 *m*/*z*.

### 2.5. Data Processing and Statistical Analysis

Non-targeted metabolomics data were imported to the Compound Discoverer 3.3 (Thermo, Boston, MA, USA) software for analysis, including peak filtering, alignment, identification, and normalization. Relevant parameters were set as follows. The retention time tolerance was 0.2 min, and the mass tolerance was 5 ppm. The signal-to-noise ratio was 3, and the minimum peak intensity was 1,000,000. The intensity tolerance was set at 30%. The results of compound identifications were further confirmed by PubChem database (https://pubchem.ncbi.nlm.nih.gov/ (accessed on 1 May 2022) according to the MS2 information. Differential metabolites were screened out based on log2 Fold Change > 1 and *p*-value < 0.05. The information is further expressed as volcano plots in the Supplementary Materials. Multivariate statistical analysis, including principal component analysis (PCA) and orthogonal projection to latent structures-discriminant analysis (OPLS-DA), was performed using SIMCA-P 14.1 software (Umetrics, Malmo, Sweden). All the data were log10 processed before importation to reduce the effect of the absolute values. The type of Pareto scaling, which considers the importance of the peaks, was selected to normalize the data. Moreover, 7-fold cross validation was used for validation. Heat maps were constructed by Multiple Experiment Viewer software (accessed on 10 June 2022). Radar diagrams were generated with Microsoft 2019. Metabolomics pathway analysis was conducted by MetaboAnalyst (https://www.metaboanalyst.ca/ (accessed on 30 June 2022).

## 3. Results and Discussion

### 3.1. Morphological Differences among Strawberry Cultivars

**Figure 1:**
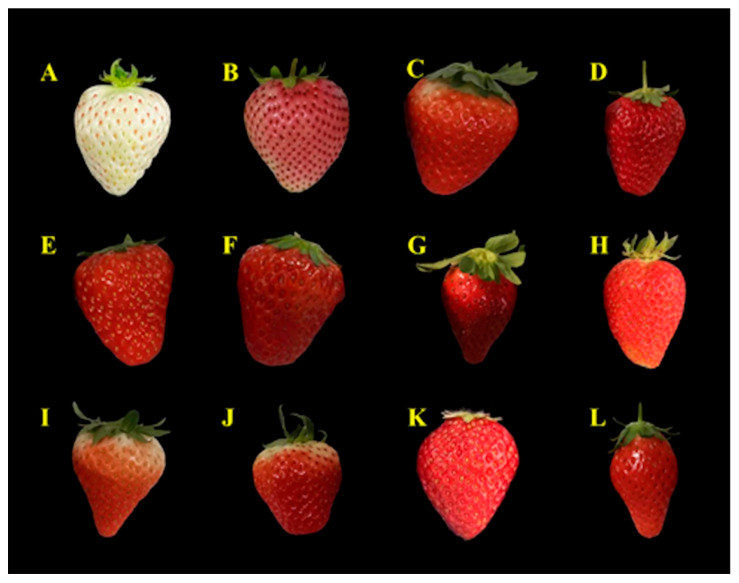
Strawberry samples used in this study. (**A**) Baiyu (BY); (**B**) Fenyu (FY); (**C**) Guangdian (GD); (**D**) Benihoppe (HY1); (**E**) Benihoppe (HY2); (**F**) Benihoppe (HY3); (**G**) Ssanta (SDH); (**H**) Kaorino (SZ); (**I**) Tongzhougongzhu (TZGZ); (**J**) Yuexiu (YX); (**K**) Tochiotome (YY); (**L**) Akihiime (ZJ).

Twelve groups of samples from 10 strawberry cultivars were analyzed. The Benihoppe variety is widely cultivated in China. Thus, three groups of samples (stands for Figure 1D–F) from the same season and region were chosen for further nutrition study. Figure 1 shows that different strawberry species were morphologically discrepant, especially in color, green pedicle, shape, and size. For example, Baiyu (Figure 1A) and Fenyu (Figure 1B) presented white and pink colors, respectively. Among the red-colored strawberry cultivars (Figure 1C–L), some were typically red (Figure 1D–H,K,L), whereas some were red and white (Figure 1C,I,J). Nevertheless, the morphologies of three groups of Benihoppe (Figure 1D–F) samples did not exhibit differences. The length and shape of green pedicles of different cultivars showed particular differences. For example, the green pedicles of Guangdian (Figure 1C), Ssanta (Figure 1G), and Tongzhougongzhu (Figure 1I) cultivars were big and long. However, Benihoppe (Figure 1D–F) and Tochiotome (Figure 1K) have short green pedicles. The mucro shape of different species showed that most of the cultivars were cuspidal, whereas the mucro shape of Benihoppe (Figure 1D–F) and Yuexiu (Figure 1J) were relatively flat. Different genotypes of strawberry cultivars presented different morphological differences from multiple aspects.

### 3.2. Identification of Metabolites in Strawberries

**Figure 2:**
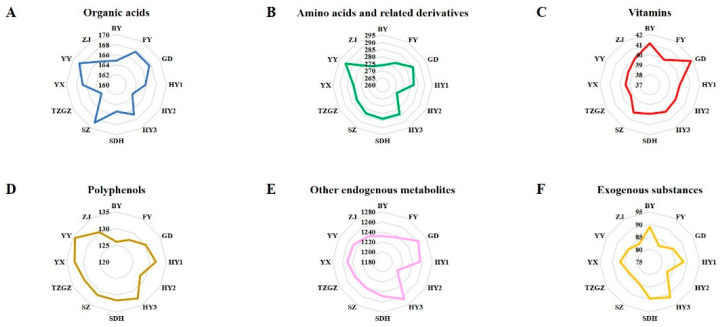
The radar maps of the identified compounds in the positive and negative ion modes. (**A**) The median value of the organic acids total content in each group; (**B**) The median value of the amino acids and related derivatives total content in each group; (**C**) The median value of the vitamins total content in each group; (**D**) The median value of the polyphenols total content in each group; (**E**) The median value of the other endogenous metabolites total content in each group; (**F**) The median value of the exogenous substances total content in each group.

**Figure 3:**
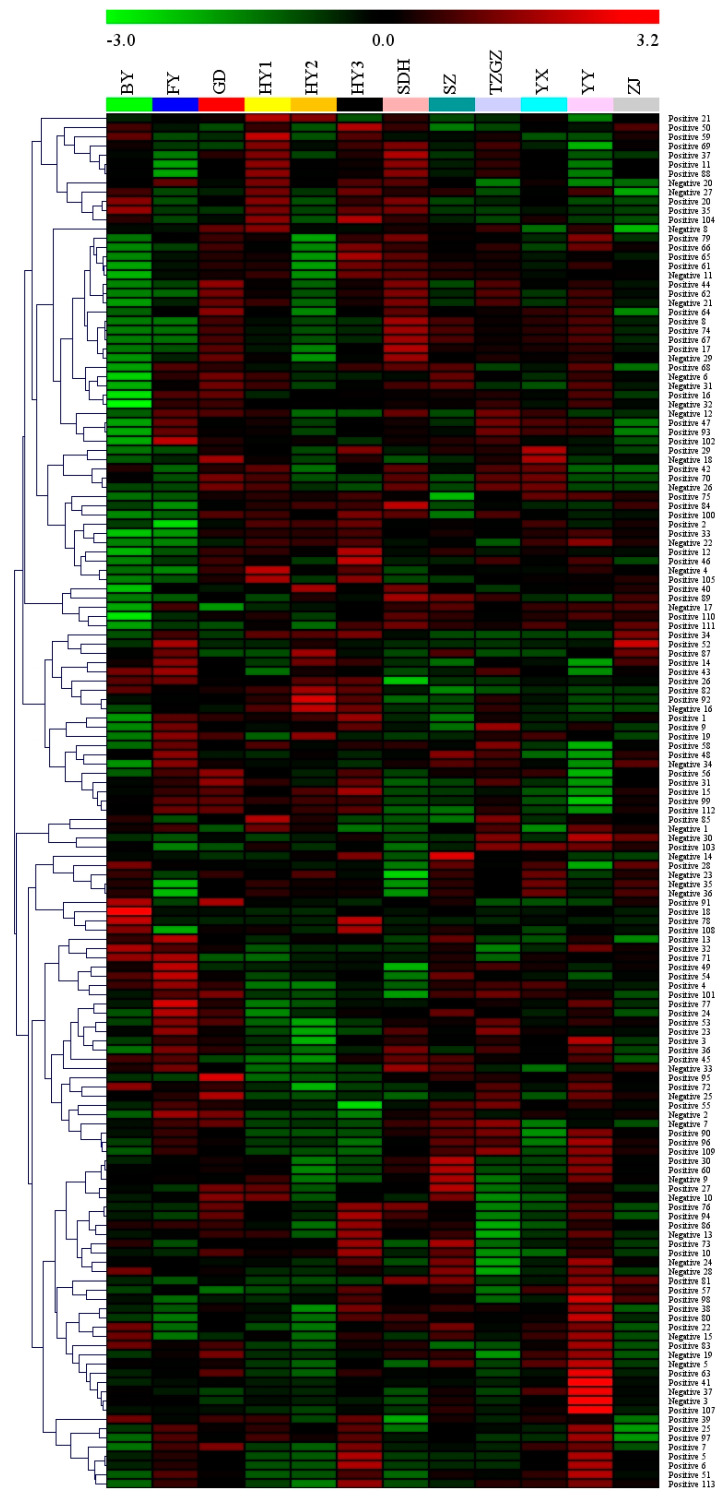
Heatmaps generated by the median contents of the identified compounds (presented as their NO. in Table S1) in strawberry samples.

The metabolites in different strawberry cultivars were detected by UHPLC-Q-Exactive Orbitrap MS in both the positive and negative ion modes. The data were then imported to the Compound Discoverer 3.3 software for further identification. A total of 5011 peaks (3932 peaks in the positive ion mode and 1079 peaks in the negative mode) were detected. All of the peaks were finally confirmed to 113 compounds (positive) and 37 compounds (negative) by the fragment information in the Pubchem library. Furthermore, eight of the identified compounds were detected in both the positive and negative ion modes. These were glutamic acid, histidine, apigenin, cytidine, phenylalanine, aspartic acid, asparagine, and O-acetylserine. Therefore, a total of 142 compounds were finally identified. Detailed information of these compounds are shown in Table S1. The representative total ion chromatograms (TIC) of QC sample in the positive and negative ion mode are shown in Figure S1. All the identified compounds in the positive and negative ion modes were classified into six categories: organic acids, amino acids and related derivatives, vitamins, polyphenols, other endogenous metabolites, and exogenous substances. The median values of the total contents of these compounds in each group are expressed as the radar maps in Figure 2. This showed different rules among the different strawberry species when summing up the compounds that belonged to the same category. For example, the median value of the organic acids total content was highest in the group of SZ. The median value of the amino acids and related derivatives and polyphenols total contents were highest in the group of YY. GD cultivar presented the highest content of vitamins. More detailed information on the total content of these six categories detected in the positive and negative ion modes is shown in Figures S2 and S3. To better understand the identified compounds in all strawberry samples, the heat map generated by the median contents was conducted (Figure 3). Red indicates a higher content, whereas green indicates a lower content. The contents of the detected compounds based on the average and median values in the cultivar of HY3 presented an overall higher level compared with other cultivars.

Our results confirmed that small molecule metabolites such as organic acids, amino acids, vitamins and polyphenols were widely detected in strawberries. Additionally, the metabolite contents varied in different strawberry cultivars. In this study, a total of eighteen organic acids were confirmed to exist in different strawberry cultivars, such as citric acid, malic acid, quinic acid and mevalonic acid. The results were consistent with previous studies of fresh strawberry samples [18]. Kinds of sugars were identified in the present study, including maltose, melezitose, and raffinose. The highest concentration of the three sugars were in the species BY, HY1, and TZGZ, respectively. This indicated the distinctions in the levels and types of sugar in different strawberry cultivars. Consumer acceptability of strawberry preserves was correlated primarily to perceived sweetness intensity and sugar content [17]. Moreover, sugar concentration is closely related to energy metabolism, which includes glycolysis, tricarboxylic acid (TCA) cycle, and others.

In our study, a total of 18 kinds of polyphenols were identified, including cyanidin 3-glucoside, peonidin 3-glucoside, cyanidin, tiliroside, et al. As can be seen from Figure 2D, the total content of polyphenols was higher in red strawberries than that in the white (BY) and pink (FY) strawberries. This may be because some of the polyphenols, such as anthocyanins, were positively correlated with strawberry color. Interestingly, a previous study revealed that compared with cultivated strawberries, wild strawberry species were found to have a higher proportion of cyanidin [19]. Chlorogenic acid, which was identified in the present study in the positive ion mode, was reportedly among the major polyphenols in strawberries [20]. The content of chlorogenic acid was found to be highest in the TZGZ cultivar of our study. Chlorogenic acid is a kind of strong antioxidant and antimicrobial agent [21]. Emerging evidence from in vitro and in vivo studies suggested that the intake of antioxidative compounds in fruits may enhance body defense against oxidative damage [4]. This finding suggests that eating more strawberries, especially the cultivars of high antioxidative compounds contents, may enhance the body’s antioxidant capacity. Equally important, vitamins, especially vitamin C (ascorbic acid), play major roles in the multiple reactions of organism metabolism. Of all the cultivars in this study, GD cultivar presented the highest content of vitamins, followed by BY and SZ. Previous study pointed out that the ascorbic acid in the cultivars of Darselect, Clery, Elianny, Diammante, and Sonata was two to three times higher than in other cultivars [22].

Amino acids were widely detected in various strawberry cultivars in the present study. This was in line with the profiled amino acids in strawberry juice in a previous study [23]. The richness of amino acids in strawberries provided health benefits, such as antimutagenicity, reduction of blood sugar, and decrease in coronary heart diseases. Moreover, amino acids played vital roles in protein synthesis and strawberry taste composition. Among all detected amino acids in the present study, glutamine was the most abundant in terms of total content in all strawberry samples, followed by pyroglutamic acid and asparagine. Aspartic acid, proline, and valine had co-pigmentation effects on individual anthocyanins in strawberry juice. The combined use of aspartic acid and proline at 105 °C may present better protective effects in anthocyanins [24]. The discrepancy of amino acids in various strawberry species may be related to their genotype and agronomic conditions, and this will lead to the different enzymatic and non-enzymatic browning phenomena during postharvest.

Besides the metabolites of the strawberry, various kinds of exogenous compounds were detected at the same time, such as chlormequat and difenoconazole. These compounds are presumed to be used during the strawberry growing process. For example, chlormequat was one of the pesticides in strawberry with the highest detection percentages, according to a previous study [25]. Similarly, although the risk of difenoconazole acute dietary exposure in strawberries among different consumer groups was less than 100%, it is necessary to conduct a comprehensive monitoring of pesticide residues in strawberry. The detection of these exogenous compounds prompted a focus on the use of the pesticides in strawberry cultivation. This will help strengthen and standardize farmers’ understanding of strawberry pest-control measures and will provide a scientific basis for the risk control of strawberry products.

### 3.3. Multivariate Statistical Analysis of the Identified Metabolites in Different Strawberry Cultivars

**Figure 4:**
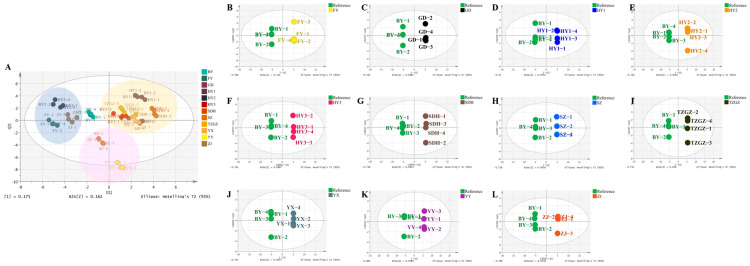
(**A**) PCA analysis result of all strawberry samples; (**B**) OPLS-DA analysis between BY and FY; (**C**) OPLS-DA analysis between BY and GD; (**D**) OPLS-DA analysis between BY and HY1; (**E**) OPLS-DA analysis between BY and HY2; (**F**) OPLS-DA analysis between BY and HY3; (**G**) OPLS-DA analysis between BY and SDH; (**H**) OPLS-DA analysis between BY and SZ; (**I**) OPLS-DA analysis between BY and TZGZ; (**J**) OPLS-DA analysis between BY and YX; (**K**) OPLS-DA analysis between BY and YY; (**L**) OPLS-DA analysis between BY and ZJ.

For better understanding of the distinctions between the endogenous metabolites among different strawberry cultivars, multivariate statistical analysis was conducted. Figure 4A presents that all the strawberry groups were separated from each other by the PCA analysis, indicating distinctions of the metabolites in different strawberry species. Moreover, pair-wise comparisons based on the OPLS-DA models are established between the BY group and other groups (R^2^ > 0.995 and Q^2^ > 0.988) in Figure 4B–L. The total separations further confirmed the diversities between the Baiyu cultivar and the other strawberry groups. Additionally, the repetitions in each group clustered well, indicating that the data exhibited good stability and reproducibility. Figure S4 showed the evaluations of the established OPLS-DA models. The 200 times permutation tests (Figure S4) showed that the original R^2^ and Q^2^ (the two right-most points) were always greater than the transposed values on the left (the left scatter points). In addition, the intercepts of Q^2^ were less than zero in all of the OPLS-DA models. These results demonstrated that all of the established OPLS-DA models were reliable and not overfit. A table of the Std. dev. values in all of the groups has been provided in Table S2.

OPLS-DA models were widely used to distinguish cultivar differences, and the process of modelling evaluation and validation was strict. Firstly, 7-fold cross validation was used. Then, R^2^Y (the explanatory ability of the model to classification variable Y) and Q^2^ (the predictability of the model) obtained after cross-validation were used to evaluate the effectiveness of the model. Finally, the replacement test was passed (permutation test), the order of Y was changed several times to obtain different random Q^2^ values, and the validity of the model was further tested (Figure S4). Similar approaches were adopted to differentiate three strawberry cultivars (Camarosa, Festival and Palomar) after GC-MS based metabolomics [26].

### 3.4. Potential Biomarkers for Distinguishing Different Strawberry Cultivars

Based on the principles of *p* value < 0.05 and log2 Fold Change > 1, the differential metabolites in the both positive and negative ion modes were screened out between the BY group and other strawberry groups. Detailed metabolite information is shown in Table S3, which lists the compounds of significant up-regulation and down-regulation of each pair-wise comparison. Of all the pair-wise comparisons with the BY group, the tochinotome cultivar (YY) may be the most different by screening out 72 differential metabolites. The representative volcano plots between the YY and BY in the positive and negative ion modes are shown in Figures S5 and S6. In addition, histidine, apigenin, cyanidin 3-glucoside and peonidin 3-glucoside were identified as potential biomarkers because they satisfied with the principles (*p* value < 0.05 and log2 Fold Change > 1) in all of the pair-wise comparisons. Taking a further look at the contents of these four potential biomarkers, they all presented higher contents in other strawberry cultivars than that in the BY group. These results suggest the differences in metabolite contents of various strawberry species, and the distinctions further determined the differentiation of taste, nutrition and even color between the BY species and other strawberry species. 

Histidine is a semi-essential amino acid for humans and other mammals. It has important functions in mood disturbance and response time [27]. During the storage of fruits and vegetables, histidine is decarboxylated by the effects of amino acid decarboxylase to produce histamine. This may be the reason for the negative effects of histidine on consumer acceptance in another study of peach fruit [28]. The content of histidine varied significantly with the differences among cultivars. For example, in a study of different fig fruit cultivars, “Masui Dauphine” had the lowest content of histidine, whereas “Banane” had the highest content [29]. The differences of histidine content in different strawberry cultivars may further determine the different sourness and storage ability of BY and other strawberries. This finding will provide a scientific basis for exploring the best postharvest treatment and storage conditions for different strawberry cultivars.

Apigenin is a kind of flavonoid with potent anti-oxidant effect. Apigenin and its various derivatives are abundant in strawberry, especially in wild strawberry, such as the native Chilean red strawberry [30,31]. In the present study, apigenin was screened out as a significant up-regulation metabolite to distinguish the BY group and other strawberry groups. The nutritional values of apigenin in different strawberry cultivars needs to be a focus of research, especially in species with high content, such as Benihoppe, Yuexiu and Tochiotome.

Cyanidin 3-glucoside and peonidin 3-glucoside are two kinds of anthocyanins with significant content differences between Baiyu and other strawberry cultivars. It is well known that cyanidin 3-glucoside was one of the major anthocyanins in strawberry. However, peonidin 3-glucoside was first identified in strawberry in 2010 [32]. They play important roles in providing attractive red color in fresh strawberry fruit [33]. The contents of these two anthocyanins were significantly lower in the Baiyu cultivar than other strawberry cultivars. This may be one of the main reasons leading to the white color of Baiyu. Recent study found that anthocyanin and co-pigment reacted with a noncovalent bond [34]. Amino acids, such as aspartic acid, can be used as co-pigment to increase the stability of anthocyanins in strawberry. Aspartic acid was also identified in different strawberry cultivars in our study. Anthocyanins a good source of nutrients in strawberry. Previous investigations have revealed antioxidant, anti-inflammatory, antihypertensive and anti-hyperlipidemic or anti-proliferative effects of strawberry anthocyanin compounds [35]. Moreover, anthocyanins are also used as dietary polyphenols in food owing to functional properties in regulating blood sugar level and lipid metabolism [36]. Therefore, eating red strawberries with high anthocyanin contents is also an effective way to increase anthocyanin dietary content.

**Figure 5:**
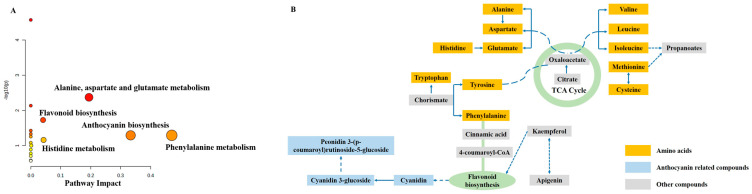
(**A**) Pathway analysis based on 14 differential endogenous metabolites that were screened out multiple times (10 and 11 times) in comparison with BY; (**B**) The relationships between amino acid metabolisms and flavonoid biosynthesis.

According to the 16 differential endogenous metabolites that were screened out multiple times (10 and 11 times) in comparison with BY, the main metabolic pathways involved are summarized in Figure 5A. Significantly different metabolic pathways were related to amino acid metabolisms, anthocyanin biosynthesis and flavonoid biosynthesis. These findings suggested that the distinctions between Baiyu and other strawberry cultivars were mainly reflected in the differences of amino acids and polyphenols. Moreover, complex interactions occurred among these amino acids, related to the tricarboxylic acid (TCA) cycle. The TCA cycle acts as an important junction station for sugars, lipids, and amino acids. A series of processes in the TCA cycle provided energy in the cell metabolism. Many kinds of organic acids are involved in the TCA cycle, including citric and malic acids, which were also identified in the present study. Related differential metabolites are summarized in Figure 5B. Phenylalanine will further participate in the flavonoid biosynthesis pathway by reacting with 4-coumaroyl-CoA. Flavonoid biosynthesis is one of the sources for synthesis of anthocyanins. Taken together, the different metabolite contents in these relevant pathways may further determine the speed and intensity of multiple reactions. This may ultimately lead to the difference in morphology, taste, and nutrition of different strawberry cultivars.

## 4. Conclusions

In the present study, we conducted a comprehensive metabolomics analysis of the metabolites in strawberry and compared the differences in their contents in 10 different cultivars. A total of 5011 peaks were detected in the positive and negative modes. They were finally identified as 142 compounds, and were further divided into six categories. The results showed that small molecule metabolites such as organic acids, amino acids, vitamins and polyphenols were widely detected in strawberries, and their contents varied with different strawberry cultivars. Multivariate analysis revealed the distinctions between the white strawberry cultivar (Baiyu) and other strawberries. Histidine, apigenin, cyanidin 3-glucoside and peonidin 3-glucoside had potential as biomarkers for distinguishing Baiyu and the other 11 strawberry groups. Pathway analysis results showed that the main metabolic pathways based on the occurrence of multiple differential metabolites were related to amino acid metabolisms, flavonoid biosynthesis and anthocyanin biosynthesis. These findings suggested that the distinctions between BY and other strawberry cultivars were mainly reflected in the differences of amino acids and polyphenols. The obtained results revealed the potential nutritional biomarkers among the ten strawberry varieties and increased our understanding of the association between strawberry cultivars and metabolite differences. Overall, the present work contributes to a scientific basis for improving strawberry nutrition in breeding and cultivation.

## Data Availability

The datasets generated for this study are available on request to the corresponding author.

## Supplementary Materials

The following supporting information can be downloaded at: https://www.mdpi.com/article/10.3390/foods12061153/s1, Figure S1: A. The representative total ion chromatogram (TIC) of strawberry sample in the positive ion mode; B. The representative total ion chromatogram (TIC) of strawberry sample in the negative ion mode. Figure S2: The radar maps of the identified compounds in the positive ion mode. Figure S3: The radar maps of the identified compounds in the negative ion mode. Figure S4: The evaluations of the metabolomics OPLS-DA models in the comparison between the BY group and other strawberry groups (permutation test, n = 200). Figure S5: The volcano plots of the pairwise comparisons with the BY group in the positive ion mode. Figure S6: The volcano plots of the pairwise comparisons with the BY group in the negative ion mode. Table S1: Detailed information of the identified compounds. Table S2: Std. dev. values in all of the groups. Table S3: Detailed metabolite information of significant up-regulation and down-regulation in each pair-wise comparison.
