# Supplementary material for: Comparative Metabolomic Analysis of the Nutritional Aspects from Ten Cultivars of the Strawberry Fruit
Source: Foods. 2023 Mar 9;12(6):1153. doi: 10.3390/foods12061153 (PMC10048718; doi:10.3390/foods12061153)
Supplement: Supplementary file 1 [file foods-12-01153-s001.zip › Supplementary Materials 1-Figures.pdf]

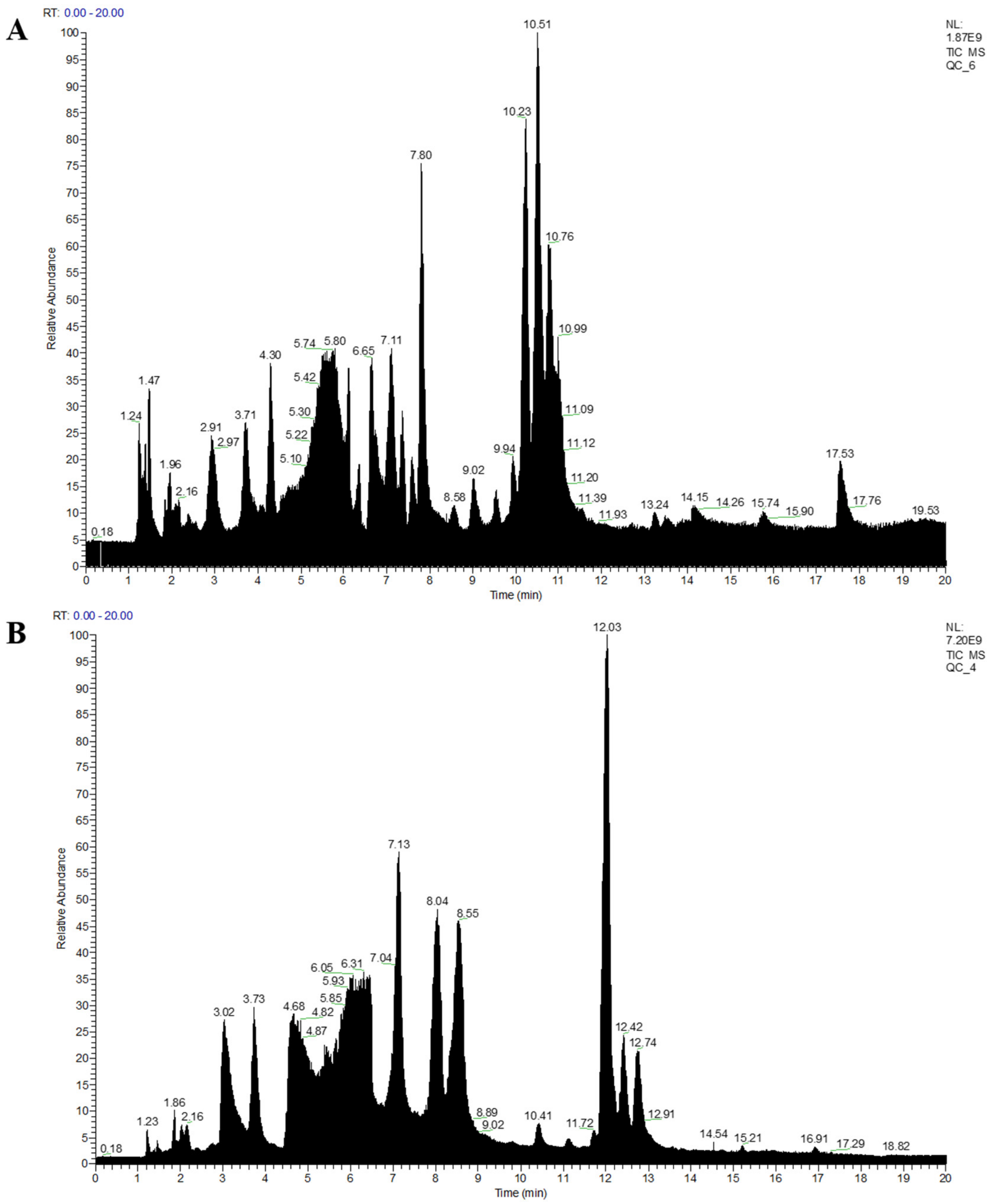

Figure S1. **(A)**. The representative total ion chromatogram (TIC) of strawberry sample in the positive ion mode; **(B)** The representative total ion chromatogram (TIC) of strawberry sample in the negative ion mode.

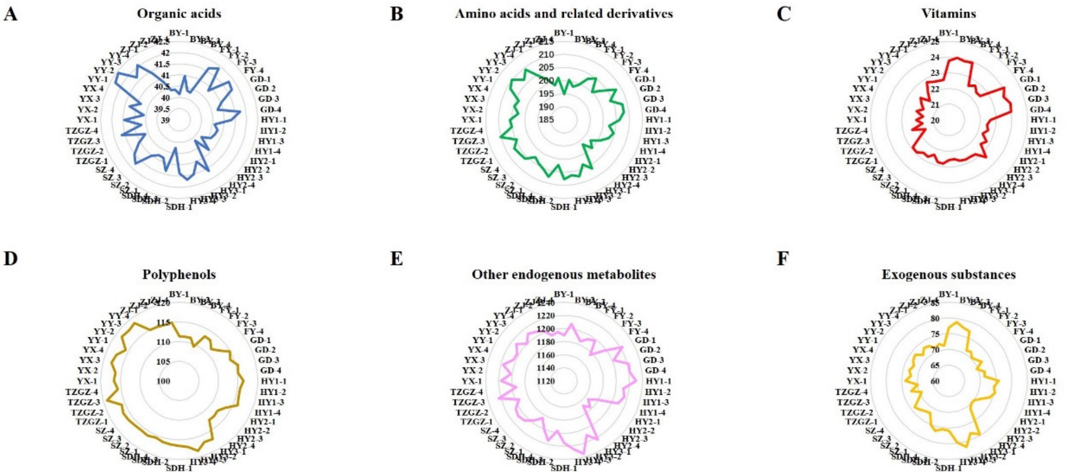

Figure S2. The radar maps of the identified compounds in the positive ion mode. **(A)** The total content of organic acids; **(B)** The total content of amino acids and related derivatives; **(C)** The total content of vitamins; **(D)** The total content of nucleosides; **(E)** The total content of other endogenous metabolites; **(F)** The total content of exogenous substances.

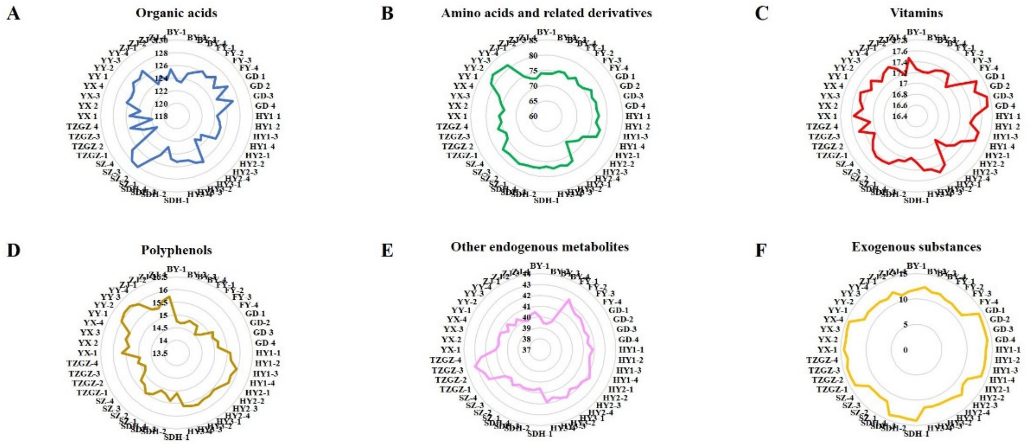

Figure S3. The radar maps of the identified compounds in the negative ion mode. **(A)** The total content of organic acids; **(B)** The total content of amino acids and related derivatives; **(C)** The total content of vitamins; **(D)** The total content of nucleosides; **(E)** The total content of other endogenous metabolites; **(F)** The total content of exogenous substances.

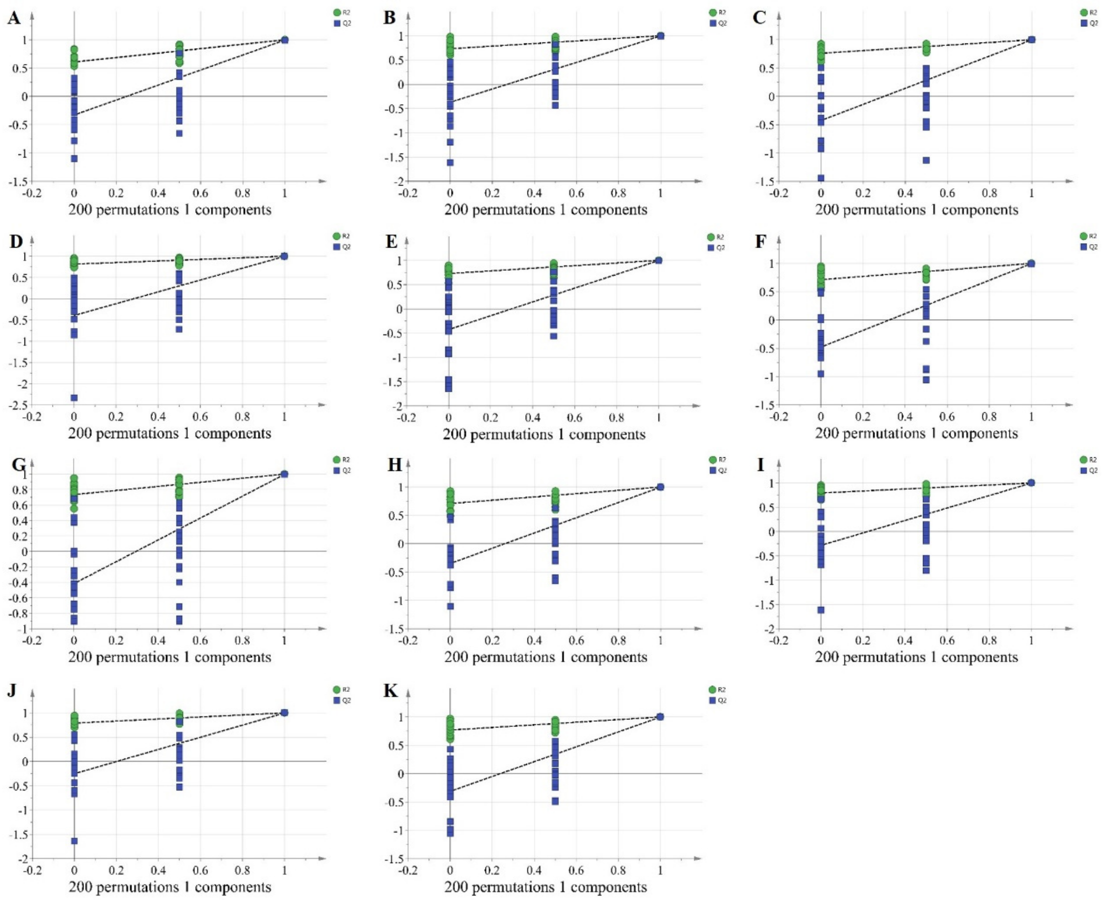

Figure S4. The evaluations of the metabolomics OPLS-DA models in the comparison between the BY group and other strawberry groups (permutation test, n=200). **(A)** BY vs FY, R^2 = (0.0, 0.616), Q^2 = (0.0, -0.338); **(B)** BY vs GD, R^2 = (0.0, 0.749), Q^2 = (0.0, -0.285); **(C)** BY vs HY1, R^2 = (0.0, 0.757), Q^2 = (0.0, -0.352); **(D)** BY vs HY2, R^2 = (0.0, 0.824), Q^2 = (0.0, -0.296); **(E)** BY vs HY3, R^2 = (0.0, 0.73), Q^2 = (0.0, -0.492); **(F)** BY vs SDH, R^2 = (0.0, 0.703), Q^2 = (0.0, -0.406); **(G)** BY vs SZ, R^2 = (0.0, 0.746), Q^2 = (0.0, -0.385); **(H)** BY vs TZGZ, R^2 = (0.0, 0.691), Q^2 = (0.0, -0.362); **(I)** BY vs YX, R^2 = (0.0, 0.781), Q^2 = (0.0, -0.372); **(J)** BY vs YY, R^2 = (0.0, 0.787), Q^2 = (0.0, -0.28); **(K)** BY vs ZJ, R^2 = (0.0, 0.772), Q^2 = (0.0, -0.261).

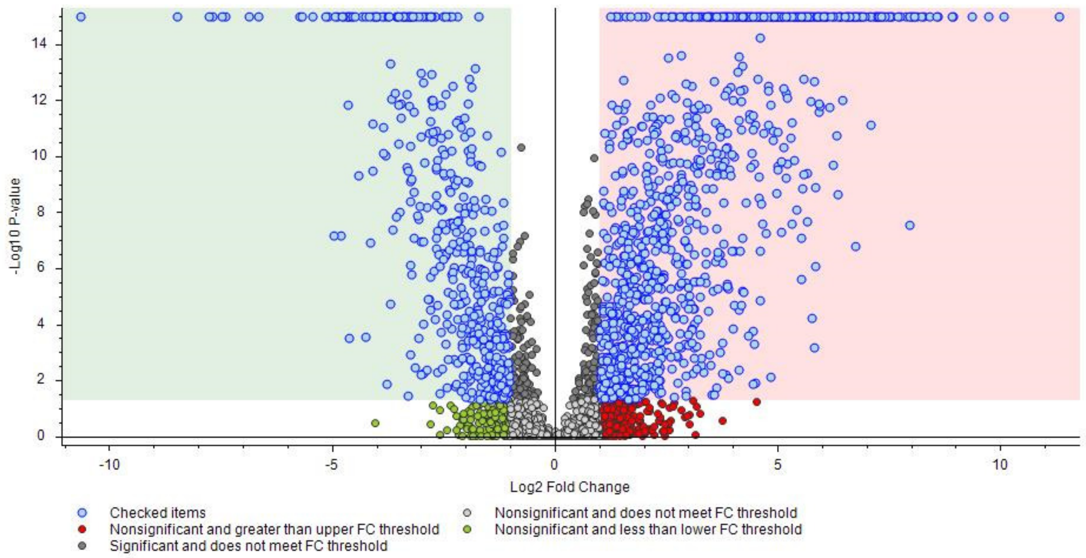

Figure S5. The volcano plot of the pairwise comparison between the YY group with the BY group in the positive ion mode.

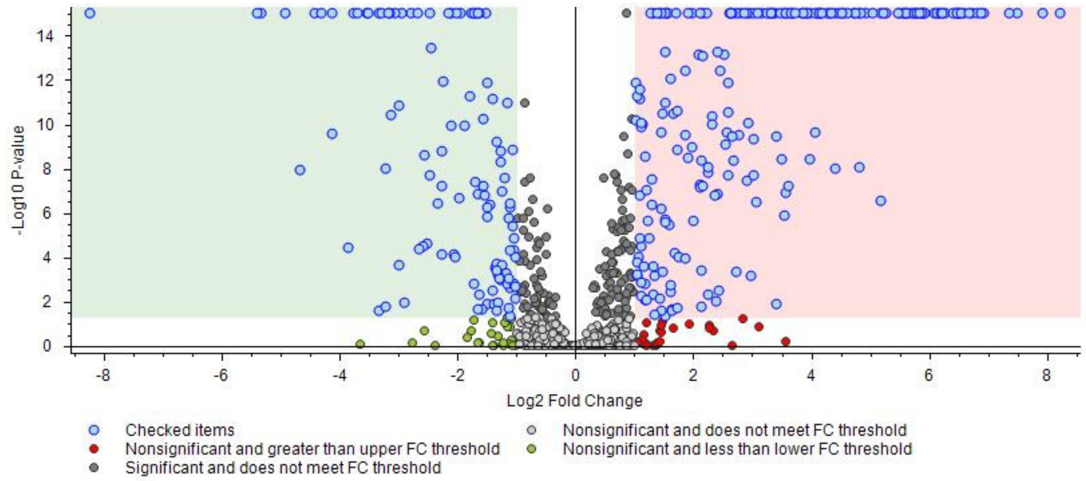

Figure S6. The volcano plot of the pairwise comparison between the YY group with the BY group in the negative ion mode.
